# A novel multi-epitope mRNA vaccine against colorectal cancer: in silico design and immune efficacy profiling

**DOI:** 10.3389/fimmu.2025.1649091

**Published:** 2025-10-23

**Authors:** Lin Wang, Xiaofei Zhou, Yu Wei, Jianping Lin

**Affiliations:** ^1^ State Key Laboratory of Medicinal Chemical Biology, Nankai University, Tianjin, China; ^2^ College of Pharmacy and Tianjin Key Laboratory of Molecular Drug Research, Nankai University, Tianjin, China; ^3^ Tianjin BioAI-global Technology Co., Ltd, Tianjin, China

**Keywords:** colorectal cancer, mRNA vaccine, epitopes, codon optimization, MHC allele

## Abstract

**Background:**

Colorectal cancer (CRC) has emerged as a growing global health challenge, while immunotherapy, particularly mRNA-based cancer vaccines, has emerged as a promising approach due to its ability to induce targeted immune responses with minimal systemic toxicity. This study aimed to design a multi-epitope mRNA vaccine targeting tumor-specific antigens (TSAs) as a cancer therapeutic regimen.

**Results:**

We chose six CRC-specific TSAs and selected their appropriate epitopes with immunoinformatic tools. In order to enhance the vaccine stability, we subsequently optimized the open reading frame (ORF) sequences, which demonstrated the highest structural stability among all evaluated approaches. Furthermore, we built a CNN model combined with RNA large language model (RNA-FM) embeddings to screen 212 candidate 5’UTR sequences and identify variants that boost the vaccine’s translational efficiency. Finally, in silico immune simulations confirmed the vaccine’s ability to elicit robust humoral and cellular immune responses.

**Conclusion:**

This study presents an in silico designed mRNA vaccine against colorectal cancer (CRC). Immune simulations demonstrated that this mRNA vaccine can elicit strong antitumor immune responses, indicating it is an effective and promising candidate that warrants further *in vitro* and *in vivo* investigations. Additionally, this work highlights the potential of in silico approaches in vaccine design and provides valuable insights for the development of effective vaccines targeting CRC.

## 1 Introduction

Colorectal Cancer (CRC) ranks as the third most prevalent malignant tumor worldwide, accounting for approximately 52,980 deaths in 2021 (1). According to global cancer research projections, an estimated 3.2 million new cases and 1.6 million deaths are anticipated by 2040, representing a 63% and 73.4% increase from 2020 levels, respectively (2). Notably, the incidence of early-onset CRC (diagnosed in individuals under 50 years of age) has risen significantly over recent decades (3). Furthermore, CRC often progresses asymptomatically during early stages, with clinical manifestations typically emerging only at advanced disease phases. These factors underscore the urgent need for novel therapeutic strategies to combat CRC (4).

Current standard CRC treatments, including surgical intervention, chemotherapy (5), and radiotherapy (6), exhibit substantial limitations. Surgery may not be feasible for all patients, particularly those with advanced-stage or metastatic disease. Chemotherapeutic agents frequently induce severe adverse effects such as nausea, vomiting, alopecia, and immunosuppression, significantly compromising patients’ quality of life (7). Moreover, the development of chemoresistance further diminishes therapeutic efficacy. Radiotherapy, while beneficial, risks damaging adjacent healthy tissues near tumor sites (8).

Recent advances in immunotherapy have positioned it as a promising alternative for cancer management (9). Among immunotherapeutic strategies, cancer vaccines have garnered considerable attention for their potential to stimulate targeted immune responses against malignant cells (10) while minimizing systemic toxicity. However, conventional vaccine platforms, including peptide-based (11), protein-based (12), and whole-cell vaccines (13), face critical challenges such as low immunogenicity, complex manufacturing processes (13), and limited capacity to simultaneously target multiple antigens (14).

The global COVID-19 pandemic has accelerated mRNA vaccine technology into a revolutionary paradigm for vaccine development. Compared to traditional approaches, mRNA vaccines offer distinct advantages: 1) Rapid design and cell-free production capabilities, crucial for addressing emerging pathogens or cancer subtypes (15); 2) Capacity to encode multiple antigens, enabling broad-spectrum immune activation against heterogeneous tumor populations (16); 3) Non-integrative nature, eliminating the risks of insertional mutagenesis associated with DNA-based vaccines (17).

**Figure 1:**
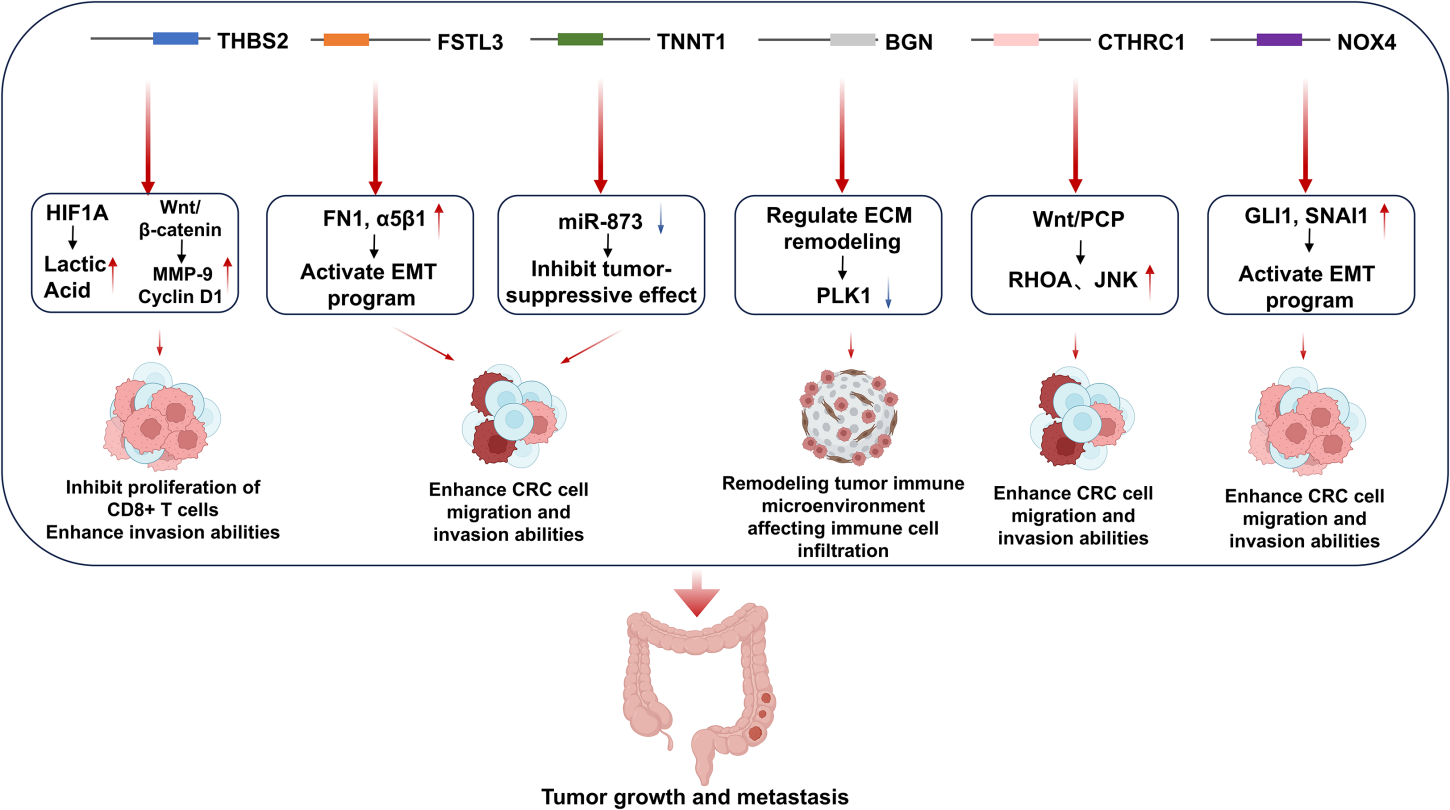
The mechanisms by which the six genes overexpressed in CRC patients regulate colorectal cancer development.

When designing effective mRNA vaccines targeting CRC, genes abnormally overexpressed in the cancer tissues of CRC patients can serve as ideal tumor-specific antigens (TSAs). According to the study by Liu et al. (18), CRC patients with high expression of six genes, *THBS2, FSTL3, TNNT1, BGN, CTHRC1, and NOX4*, exhibit shorter overall survival (OS) and shorter recurrence-free survival (RFS). Additionally, the expression levels of these six genes are associated with the infiltration levels of antigen-presenting cells (APCs) and T lymphocytes. Thus, in this study, we selected these six genes as ideal CRC-TSAs for mRNA vaccine development. Mechanistically, *THBS2* promotes CRC metastasis by modulating the WNT/β-catenin signaling pathway while suppressing antitumor immunity through HIF1A/lactate/GPR132 axis interactions (19). *FSTL3* drives epithelial-mesenchymal transition (EMT) via fibronectin1/α5β1 interactions and serves as a biomarker for extracellular matrix remodeling in CRC diagnosis (20). *TNNT1* regulates EMT processes through miR-873-mediated mechanisms and functions as a prognostic indicator for colon adenocarcinoma (21). *BGN* has been reported to exert a significant impact on CRC cell proliferation, cell cycle progression, apoptosis, invasion, and migration (22). Previous studies have shown that knockout of *BGN* can inhibit the proliferation and migration of CRC cells (23). *CTHRC1* enhances the proliferation and invasiveness of human CRC cells by activating the Wnt/planar cell polarity (PCP) signaling pathway (24). *NOX4* regulates the expression of genes related to cancer cell biological behaviors, thereby promoting CRC cell proliferation, inhibiting apoptosis, and enhancing cell migration and invasion (25). The schematic diagram illustrating how the six aforementioned genes regulate the progression of colorectal cancer (CRC) is shown in **Figure 1**.

This study aims to develop and evaluate a novel mRNA vaccine targeting CRC-specific TSAs to enhance antitumor immunity, potentially offering a therapeutic alternative for CRC patients. Through rational antigen selection and advanced bioengineering approaches, we seek to address current limitations in CRC management while leveraging the unique advantages of mRNA vaccine technology.

## 2 Material and methods

### 2.1 Retrieval of tumor-specific protein sequences

The UniProt database (https://www.uniprot.org/) was utilized to acquire amino acid sequences of the six target proteins using their respective accession numbers (1): THBS2 (P35442), (2) FSTL3 (P95633), (3) TNNT1 (P13805), (4) BGN (P21810), (5) CTHRC1 (Q96CG8), and (6) NOX4 (Q9NPH5).

### 2.2 Prediction of immune cell epitopes

#### 2.2.1 B-cell epitope prediction

B-cell epitopes were predicted using the ABCPred web server (https://webs.iiitd.edu.in/raghava/abcpred/), a machine learning platform trained on linear B-cell epitope data from the BCIPEP database. Parameters were configured with an epitope length of 16 amino acid residues, a prediction threshold of 0.5, and an activated overlap filter to ensure sequence exclusivity (26).

#### 2.2.2 Cytotoxic T lymphocyte epitope prediction

HLA class I-restricted epitopes were identified through the NetMHCPan 4.1 EL algorithm on the IEDB Analysis Resource (http://tools.iedb.org/mhci/). Predictions employed default reference sets of HLA alleles, with epitopes ranked by percentile binding scores.

#### 2.2.3 Helper T lymphocyte epitope prediction

HLA class II-binding epitopes were determined using the NetMHCIIPan 4.1 EL method via the IEDB server (http://tools.iedb.org/mhcii/). Epitope candidates were similarly prioritized based on predicted binding affinities (27).

### 2.3 Prediction of epitopes’ antigenicity, allergenicity and toxicity

Following the prediction of linear B-cell epitopes (LBL), cytotoxic T lymphocyte (CTL), and helper T lymphocyte (HTL) epitopes, we systematically evaluated the immunobiological properties of candidate epitopes. Antigenicity was assessed using the VaxiJen v3.0 web server (http://www.ddg-pharmfac.net/vaxijen/), configured with the tumor protein antigen model at a threshold score of 0.5. This machine learning platform employs alignment-free algorithms trained on bacterial, viral, and tumor antigen datasets to predict whole-protein immunogenicity. Allergenicity screening was performed through AllerTop v2.1 (https://www.ddg-pharmfac.net/allertop_test/), which utilizes amino acid propensity scales and auto-cross covariance transformation for epitope safety evaluation. Toxicity predictions were conducted via ToxinPred (https://webs.iiitd.edu.in/raghava/toxinpred/), employing support vector machine models trained on toxic/non-toxic peptide datasets. Epitopes demonstrating strong antigenicity (VaxiJen score ≥0.5), non-allergenicity, and non-toxicity were retained for downstream analyses.

### 2.4 Population coverage of epitopes

Geographical and environmental factors drive substantial diversity in human leukocyte antigen (HLA) alleles across global populations. To evaluate the epidemiological relevance of our vaccine candidate, we conducted global population coverage analysis using the Immune Epitope Database (IEDB) Population Coverage Calculation Tool (http://tools.iedb.org/population/). This platform calculates cumulative coverage probabilities for selected HLA class I (CTL) and class II (HTL) epitopes based on their binding affinities to region-specific HLA allele distributions across 16 geographical regions. The analysis integrated frequency data of HLA-A, -B, -C, and -DRB1 alleles from ethnically diverse populations to estimate potential vaccine efficacy thresholds.

### 2.5 Molecular docking between T-lymphocyte epitopes and MHC alleles

To evaluate the binding affinity between selected T lymphocyte epitopes and their corresponding major histocompatibility complex (MHC) class I alleles, we performed systematic molecular docking simulations. MHC-I crystal structures were retrieved from the RCSB Protein Data Bank (PDB). Structural preprocessing involved removal of water molecules and non-essential ligands using PyMOL v2.5.7 (28). Epitope sequences underwent 3D conformational modeling via PEP-FOLD 3.5 server (https://bioserv.rpbs.univ-paris-diderot.fr/services/PEP-FOLD3/), followed by complex assembly with MHC-I proteins. Energy minimization was conducted using Rosetta’s Relax protocol (v3.13) to optimize molecular geometries (29). Finally, epitope-MHC docking simulations were executed in local refinement mode through RosettaDock, employing Monte Carlo minimization algorithms to sample low-energy binding conformations.

### 2.6 Screening of 5’UTR

The 5’UTR sequence in particular is a major determinant of translation efficiency and thus an intriguing target for engineering (30). The ribosomal 43 S pre-initiation complex (PIC) scans the 5’UTR in the 5’-to-3’ direction until a start codon is found. Therefore, 5’UTRs can affect translation by capturing PICs prematurely via upstream start codons (uAUGs) and ORFs (uORFs) (31). To identify 5′-UTR variants with enhanced translational efficiency, we employed RNA-FM (32), a pretrained large language model, to generate embeddings for 83,919 synthetic human 5′-UTRs of 75 distinct lengths from the combinatorial library generated by Sample et al. (33). The convolutional neural network (CNN) architecture was constructed based on Sample et al. to screen candidate 5′-UTRs. The CNN model was constructed as three 1D convolutional layers with 120 filters and a ReLU activation for each layer. The third convolution layer output one channel, which was fed into two fully-connected layers with one output node as the final prediction. The inputs of the model were RNA-FM embeddings (640 dim). In this study, the data was partitioned into a training set and a validation set with a ratio of 8:2. This partitioning strategy aimed to ensure sufficient data for model training while enabling effective validation of the model’s generalization ability.

Our 5′-UTR candidate pool integrates four distinct sources: 1) 212 variable-length sequences from Chu et al. (34), 2) 8 Kozak sequence-containing variants from Li et al. (35), 3) The 5′-UTR of human α-globin genes, and 4) the 5′-UTR of BNT162b2(SARS-CoV-2 vaccine, from Pfizer/BioNTech) (36).

### 2.7 Rational design of mRNA vaccine construct

A potent mRNA vaccine construct requires systematic integration of five critical components (1): an open reading frame (ORF) encoding antigenic elements (2), the Untranslated Regions (UTRs) flanking the coding regions, (3) a Kozak sequence incorporating the start codon (GCCACCAUGG) to enhance translational initiation (37), (4) functionally optimized linkers, and (5) regulatory termination signals. The proposed construct features a 5’→3’ architecture comprising a modified 5′m^7^GCap structure, followed by an optimized 5’ untranslated region (5’UTR) and Kozak sequence to maximize ribosomal engagement. The ORF initiates with a signal peptide for intracellular trafficking, connected via an EAAAK rigid linker to helper T lymphocyte (HTL) epitopes interconnected through GPGPG spacers that maintain domain autonomy. This HTL cluster transitions via KK linkers to linear B-cell epitopes (LBL), followed by AAY-linked cytotoxic T lymphocyte (CTL) epitopes strategically positioned upstream of an MHC class I trafficking domain (MITD). The MITD sequence enhances immunogenicity through dual mechanisms: 1) optimizing antigen presentation efficiency via endosomal targeting motifs, and 2) directing vaccine components to antigen-processing compartments. Epitope segregation through GPGPG, KK, and AAY linkers ensures proper conformational folding while preserving immunological functionality. The construct terminates with a UAA stop codon, a stabilizing 3’ untranslated region (3’UTR), and a 120-nucleotide poly(A) tail to ensure mRNA stability and translational fidelity. This multi-layered design leverages structural bioengineering principles to balance epitope accessibility, intracellular trafficking efficiency, and immune activation capacity.

### 2.8 Prediction of antigenicity, allergenicity, toxicity and physicochemical properties of the vaccine construct

Following vaccine sequence assembly, systematic bioinformatic validation was performed to assess four critical properties: (1) antigenicity, (2) allergenicity, (3) toxicity, and (4) physicochemical stability. Antigenicity prediction employed dual machine learning platforms—VaxiJen v3.0 (threshold: 0.5; tumor antigen model) and ANTIGENpro (SCRATCH Protein Predictor suite)—using the translated amino acid sequence excluding trafficking/processing elements (tPA and MITD domains). Allergenicity screening was conducted through AllerTop v2.1 (https://www.ddg-pharmfac.net/allertop_test/) using default parameters. Toxicity profiling utilized ToxinPred (https://webs.iiitd.edu.in/raghava/toxinpred/), implementing SVM models trained on experimentally validated toxic peptides. Physicochemical characterization was performed via the ProtParam tool (https://web.expasy.org/protparam/), quantifying six essential parameters: amino acid composition, molecular weight, theoretical isoelectric point (pI), instability index (II), aliphatic index (AI), and grand average of hydropathicity (GRAVY). This multi-platform validation strategy ensures structural integrity while confirming immunological safety and biophysical stability of the vaccine candidate.

### 2.9 In silico immune simulation

To predict the immunogenic potential of the mRNA vaccine construct in humans, we conducted in silico immune simulations using the C-IMMSIM immunoinformatics platform (https://kraken.iac.rm.cnr.it/C-IMMSIM/). The vaccination regimen was configured to follow the recommended dosage schedule of current vaccines: three 1,000-unit doses administered at time-steps 1, 84 (3 weeks), and 168 (6 weeks). All parameters were maintained at default settings. Dynamic simulation outputs quantified key immunological metrics: 1) Antigen-specific lymphocyte proliferation rates, 2) Cytokine production profiles, 3) Memory cell differentiation kinetics, and 4) Antibody titer trajectories. This computational framework enables systematic evaluation of the vaccine’s capacity to elicit coordinated humoral and cellular immune responses while predicting long-term immunological memory formation.

### 2.10 Codon optimization and sequence refinement

Leveraging the degeneracy of the genetic code, we implemented a multi-algorithm optimization strategy to enhance translational efficiency of the mRNA vaccine construct. Five codon optimization methods, including LinearDesign (38), were systematically applied to resolve synonymous codon conflicts while maximizing expression potential. Post-optimization sequences underwent rigorous biophysical characterization through three key metrics: 1) Minimum free energy (MFE) of mRNA secondary structures predicted by RNAfold (http://rna.tbi.univie.ac.at/cgi-bin/RNAWebSuite/RNAfold.cgi), 2) GC content analysis (optimal range: 45-55%), and 3) Codon Adaptation Index (CAI) calculations relative to human codon usage tables. This combinatorial approach achieves dual objectives: (a) elimination of cryptic splice sites and ribosomal drop-off sequences through codon bias correction, and (b) stabilization of mRNA architecture via thermodynamic optimization of folding patterns.

### 2.11 Secondary structure prediction of the designed mRNA vaccine

The RNAfold web server was employed to predict the secondary structure of the mRNA vaccine construct using McCaskill’s partition function algorithm (39). This computational framework calculates the minimum free energy (MFE) conformation through dynamic programming-based thermodynamic modeling of RNA folding pathways. The analysis yielded two critical outputs: 1) the MFE-optimized secondary structure visualization, and 2) quantitative thermodynamic stability metrics (ΔG in kcal/mol). Subsequent evaluation of mRNA structural features focused on identifying persistent stem-loop formations and regions of high base-pairing potential that might impede ribosomal scanning. This characterization enabled rational design optimization to balance thermodynamic stability (ΔG < -300 kcal/mol) with translational efficiency through strategic codon rearrangement in unstable regions (MFE > -150 kcal/mol). The refined mRNA architecture demonstrates enhanced resistance to endonucleolytic degradation while maintaining optimal ribosomal accessibility-critical parameters for ensuring structural integrity and sustained antigen expression in human physiological conditions.

### 2.12 Molecular docking of the designed vaccine

To investigate the vaccine construct’s innate immune activation potential, we performed protein-protein docking between predicted vaccine epitopes and human Toll-like receptor 3 (TLR3; PDB ID 1ZIW) and Toll-like receptor 4 (TLR4; PDB ID 3FXI) using the ClusPro 2.0 server (https://cluspro.org/). This rigid-body docking platform employs a hierarchical protocol combining fast Fourier transform (FFT) correlation approaches with Monte Carlo minimization to sample >10^9^ possible binding conformations.

## 3 Results

### 3.1 B-cell epitope prediction and selection

B-cell epitopes were predicted for the amino acid sequences of THBS2, FSTL3, TNNT1, BGN, CTHRC1, and NOX4 using the ABCPred web server. For each protein, the top five scoring epitopes were initially retained based on prediction rank. Subsequent refinement employed a tripartite filtering strategy: 1) Antigenicity validation via VaxiJen v3.0 (threshold: 0.5; tumor antigen model), 2) Allergenicity screening using AllerTop v2.1, and 3) Toxicity profiling through ToxinPred. A total of six epitopes demonstrating strong antigenicity (VaxiJen score ≥0.5), non-allergenicity, and non-toxicity were selected for final inclusion (**Supplementary Table S1**). This stringent selection protocol ensures exclusive retention of epitopes with optimal immunogenic potential while mitigating risks of hypersensitivity or cytotoxic responses.

### 3.2 Prediction and estimation of the CTL epitopes

Potential cytotoxic T lymphocyte (CTL) epitopes across the six target proteins were predicted using the MHC-I Binding Predictions tool within the IEDB Analysis Resource (http://tools.iedb.org/mhci/). Predicted epitopes were subsequently filtered through a triaxial validation pipeline: 1) Antigenicity scoring via VaxiJen v3.0 (threshold ≥0.5), 2) Allergenicity assessment using AllerTop v2.1, and 3) Toxicity profiling through ToxinPred. Epitopes were prioritized based on combined metrics: percentile binding rank (<1.0), antigenicity score (>0.7), and evolutionary conservation index (>0.8 in ConSurf analysis). Twelve high-affinity epitopes residing in phylogenetically conserved regions were ultimately selected for vaccine incorporation (**Supplementary Table S1**), ensuring broad HLA coverage and variant-resistant immunogenicity.

### 3.3 Prediction and estimation of the HTL epitopes

Potential helper T lymphocyte (HTL) epitopes were identified through comprehensive analysis of the six target proteins using the IEDB MHC-II Binding Predictions tool (http://tools.iedb.org/mhcii/) with NetMHCIIPan 4.1 algorithm. Following rigorous triaxial screening—antigenicity assessment (VaxiJen v3.0 score ≥0.5), allergenicity profiling (AllerTop v2.1), and toxicity evaluation (ToxinPred), 18 epitopes residing in phylogenetically conserved domains (ConSurf conservation score >0.85) were prioritized for vaccine inclusion (**Supplementary Table S1**). After calculation and filtering, 18 selected HTL epitopes could induce Th1/Th2-polarized cytokine responses (IFN-γ, IL-4, IL-10) through TCR-MHC II complex stabilization, thereby amplifying both humoral and cellular arms of vaccine-induced immunity via cognate T-B lymphocyte collaboration.

### 3.4 Population coverage analysis

The worldwide epidemiological relevance of the vaccine construct was evaluated through HLA allele population coverage analysis using the IEDB Population Coverage Calculation Tool (http://tools.iedb.org/population/). The 30 incorporated HLA alleles demonstrated differential coverage across 16 geographical regions, with peak efficacy observed in Europe (89.13%) and North America (84.69%) (**Table 1**). Comparatively reduced coverage rates were identified in Central America (15.29%), South America (52.23%), and Northeast Asia (52.38%), reflecting regional disparities in HLA allele distribution patterns (details in **Supplementary Figure S1**).

**Table 1:** The population coverage of each epitope and its corresponding HLA allele in the mRNA vaccine.

population/area	coverage	average_hit	pc90
Central Africa	63.33%	1.32	0.27
Central America	15.29%	0.22	0.12
East Africa	70.10%	1.49	0.33
East Asia	63.42%	1.32	0.27
Europe	89.13%	2.94	0.92
North Africa	69.31%	1.66	0.33
North America	84.69%	2.43	0.65
Northeast Asia	52.38%	0.91	0.21
Oceania	60.96%	1.04	0.26
South Africa	55.74%	1.32	0.23
South America	52.23%	1.02	0.21
South Asia	71.86%	1.8	0.36
Southeast Asia	54.27%	0.86	0.22
Southwest Asia	55.18%	1.18	0.22
West Africa	67.90%	1.54	0.31
West Indies	77.19%	1.99	0.44
Average	62.69	1.44	0.33
Standard deviation	16.29	0.63	0.19

Average hit means the average number of epitope hits/HLA combinations recognized by the population, and the pc90 means minimum number of epitope hits/HLA combinations recognized by 90% of the population.

### 3.5 Molecular docking between HLA alleles and the selected T-lymphocyte epitopes

**Figure 2:**
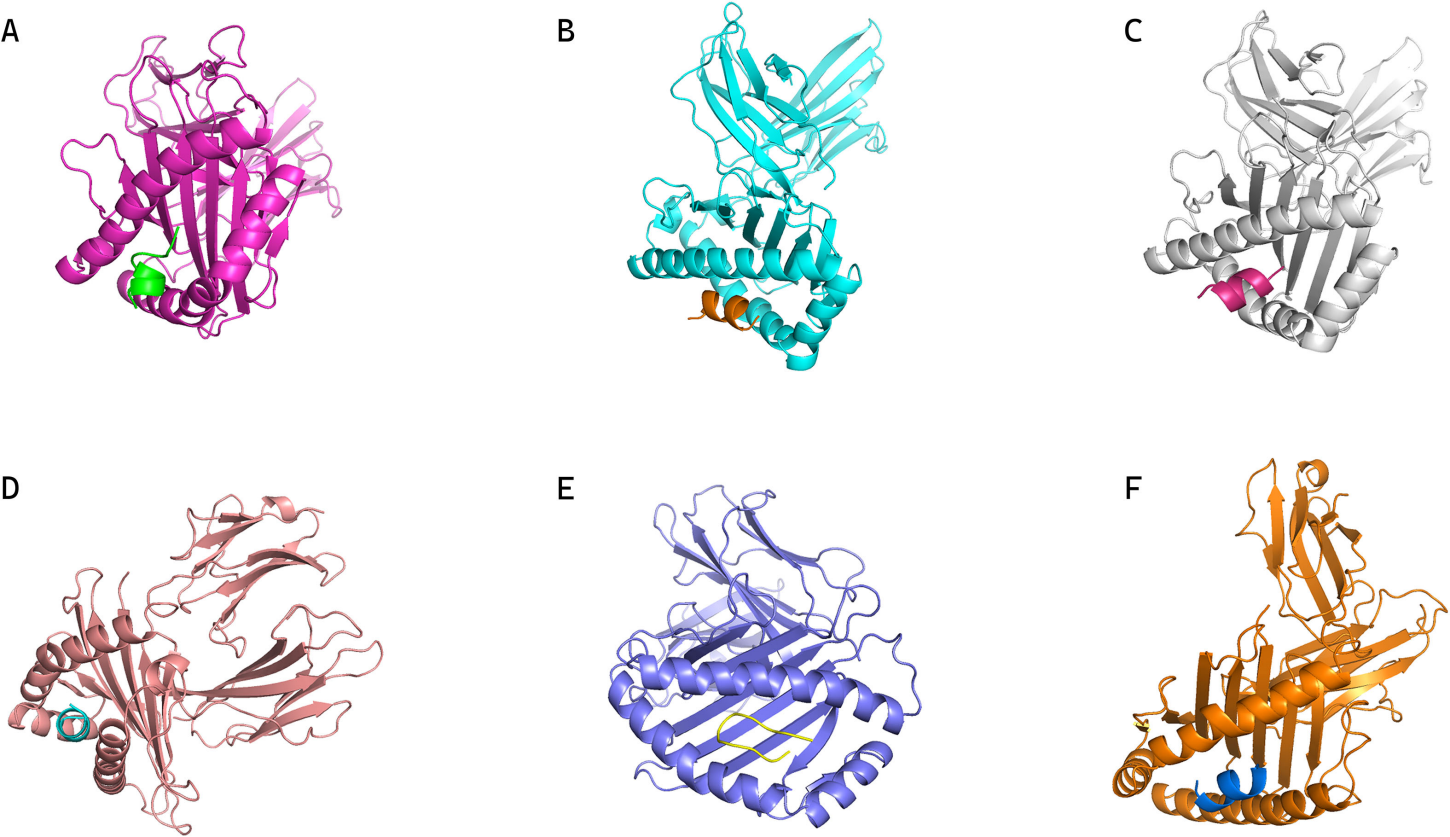
Conformations of epitopes bound to their corresponding MHC alleles from molecular docking. **(A)** Conformation of HLA-B35:01 in complex with epitope peptide DPDSVTPTY. **(B)** Conformation of HLA-B07:02 in complex with epitope peptide IPKGKQKAQL. **(C)** Conformation of HLA-A03:01 in complex with epitope peptide VMYRGRCRK. **(D)** Conformation of HLA-B35:01 in complex with epitope peptide SPFEESLNY. **(E)** Conformation of HLA-B57:01 in complex with epitope peptide RVSNDNQFLW. **(F)** Conformation of HLA-A01:01 in complex with epitope peptide MSDTEEQEY, which shows optimal binding (lowest I_sc and complete accommodation within the HLA binding groove) as supported by docking metrics.

Through the previous prediction and screening steps, 30 lymphocyte epitopes and their corresponding HLA alleles were identified (**Supplementary Table S2**), and six of these epitope-allele pairs underwent molecular docking simulations using Rosetta’s LocalDock module. Crystal structures of selected HLA class I molecules (PDB IDs: 8EMF, 7LG0, 7L1C, 4LNR, 5VUD, 6MPP) were energy-minimized to remove steric clashes (<0.3 Å RMSD deviation). Docking metrics (**Supplementary Table S3**) revealed two critical energy parameters: (1) Total_Score, predominantly reflecting monomeric folding energy, and (2) Interface_Score (I_sc), quantifying interaction energy across the binding interface. As emphasized in Rosetta documentation, I_sc provides superior predictive value for epitope-HLA binding stability. The MSDTEEQEY epitope demonstrated optimal binding with HLA-A*01:01, achieving the lowest I_sc value (-115.51 REU) and complete structural accommodation within the HLA binding groove (**Figure 2F**).

### 3.6 Screening of 5’-UTR

Our CNN model was trained and validated over 50-epoch using 83,919 human 5′-UTR sequences (spanning 75 distinct lengths) from Sample et al.’s library. Optimal regression performance was achieved at epoch 15, yielding an R² value of 0.844 with corresponding error metrics (mean squared error [MSE] = 0.307, mean absolute error [MAE] = 0.397, root mean squared error [RMSE] = 0.554). External prediction conducted on 222 literature-curated 5′-UTR sequences identified 10 top-performing candidates, with complete rankings detailed in **Table 2**. The highest-ranked variant (sequence: GGGATCTTATTCCACCTTCTGAAGCTTCTGTCGAACCAGTTGTAAGGAGA) was ultimately selected as the 5′-UTR component for our CRC mRNA vaccine construct.

**Table 2:** Predictions of relative ribosome load for 222 literature-curated 5’-untranslated region (5’-UTR) sequences using the RNA-FM embedding combined with convolutional neural network (CNN) model.

5’-UTR	length	predict_mrl
GGGATCTTATTCCACCTTCTGAAGCTTCTGTCGAACCAGTTGTAAGGAGA	50	0.985138357
CGTGAAGGCAAAGAGAACACGCTGCAAAAGGCTTTCCAAGAATCCTCGAC	50	0.953278959
GAATTATCAGAAATACTTTATAGTTATCAAAAATTCTAAAGAAAAAGGCC	50	0.953139484
GAGAGATCCAGCCTCTCAAACATCCAGCAGAGAGACCATAGGCTGCTGCA	50	0.927385569
ACACTTTCTTCTGACATAACAGTGTTCACTAGCAACCTCAAACAGACACC	50	0.927229881
CTGGAGTCTCCGCGGGCAGATCTCATATTTTGGATTCTGGATATATTATA	50	0.927137017
GGTAGTTCGGATTACTTCTTTAAGTCTCTTTTCTCTTTTTTCGCGCAAAA	50	0.922530532
GCAGTTGGGCAGCGGTTTTACCTCCATTTTGAGACCAGACAACTGGACTC	50	0.920656741
ACATTTGCTTCTGACACAACTGTGTTCACTAGCAACCTCAAACAGACACC	50	0.884883046
GAAGCCAACAAGAATTTGAGAACTGTAAATACCAAGCCTTGAAAGGGACC	50	0.88337332

### 3.7 Vaccine construct design

**Figure 3:**
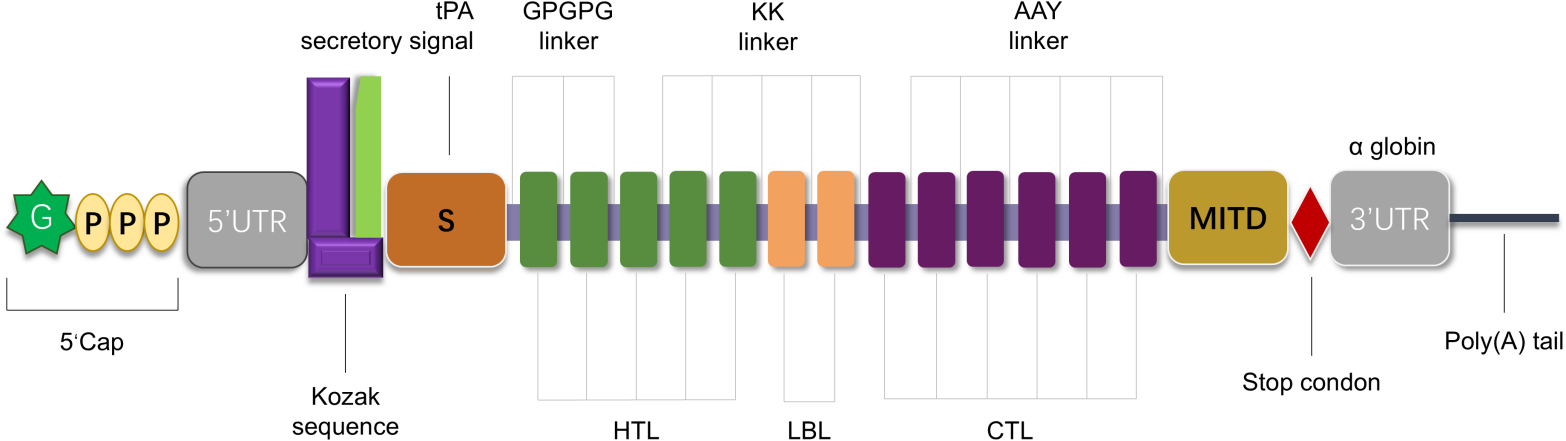
Schematic representation of the designed mRNA vaccine construct. The construct is organized from the 5′ to 3′ terminus, including: 5′ m^7^G cap; 5′ untranslated region (5′UTR) and Kozak sequence; tissue plasminogen activator (tPA) secretory signal peptide; multiple epitopes [helper T cell epitopes (HTL, green rectangles), potential B cell epitopes (LBL, orange rectangles), and cytotoxic T lymphocyte epitopes (CTL, purple rectangles)] connected by GPGPG linkers, KK linkers, or AAY linkers to ensure appropriate spatial distribution and folding; MITD sequence; stop codon; 3′ untranslated region (3′UTR); Poly **(A)** tail.

The mRNA vaccine construct was proposed to be arranged from the N to C terminus in the following order:5′m^7^GCap, 5′UTR, Kozak sequence, Signal peptide (tPA)–GPGPG Linker–LLQVVYLHSNNITKV–GPGPG Linker–TTLLDLQNNDISELR–GPGPG Linker–KISKIHEKAFSPLRK–GPGPG Linker–RDGFKGEKGECLRES–GPGPG Linker–GRDGFKGEKGECLRE–GPGPG Linker–MNSTINIHRTSSVEG–GPGPG Linker–FVSSMGSGNPAPGGV–GPGPG Linker–GFVSSMGSGNPAPGG–GPGPG Linker–VSSMGSGNPAPGGVC–GPGPG Linker–IQKIIGEKYHALNSR–GPGPG Linker–KPAEFTQHKFVKICM–GPGPG Linker–RWKLLFDEIAKYNRG–GPGPG Linker–NCPYVHNPAQIDTDN–GPGPG Linker–DNCPYISNANQADHD–GPGPG Linker–TAQLKQDGKSRGTLL–GPGPG Linker–SEKFDLMAKLKQQKY–GPGPG Linker–FDLMAKLKQQKYEIN–GPGPG Linker–QQRFRTEKERERQAK–KK Linker–DEEASGADTSGVLDPD–KK Linker–ACCQRWYFTFNGAECS–KK Linker–PQSCVVDQTGSAHCVV–KK Linker–IGRPRWKLLFDEIAKY–KK Linker–SGTQQRGRSCDVTSNT–KK Linker–YNRISHAQKFRKGAGK–AAY Linker–DPDSVTPTY–AAY Linker–KLQKLYISK–AAY Linker–KQKAQLRQR–AAY Linker–IPKGKQKAQL–AAY Linker–MRPGAPGPLW–AAY Linker–VMYRGRCRK–AAY Linker–SPFEESLNY–AAY Linker–QANFPQTWLW–AAY Linker–SVDFSGTFY–AAY Linker–RVSNDNQFLW–AAY Linker–MSDTEEQEY–AAY Linker–KEEEELVAL–AAY Linker–MITD sequence–Stop codon–3′UTR–Poly(A) tail. The schematic diagram of the vaccine construct is shown in **Figure 3**, where key components (signal peptide, HTL/LBL/CTL epitope clusters, linkers, and regulatory elements) are color-coded to clarify the 5′→3′ architecture.

### 3.8 Evaluation of antigenicity, allergenicity, toxicity and physicochemical properties of the vaccine construct

Following assembly of the complete mRNA vaccine construct, we systematically evaluated the translated polypeptide sequence through three critical safety parameters: (1) antigenicity prediction using VaxiJen v3.0 (threshold ≥0.5; tumor antigen model) and ANTIGENpro (SCRATCH suite), (2) allergenicity screening via AllerTop v2.1, and (3) toxicity profiling through ToxinPred. Physicochemical characterization was performed using the ExPASy ProtParam server (https://web.expasy.org/protparam/), quantifying six biostability metrics: molecular weight (54603.7), theoretical pI (9.27), instability index (47.94), aliphatic index (60.57), grand average of hydropathicity (GRAVY: -0.776), and thermal stability (*in vitro* half-life >30 hours at 50 °C). As summarized in **Table 3**, the construct demonstrated strong antigenicity (VaxiJen: 0.7284; ANTIGENpro: 0.93), non-allergenicity, and non-toxicity.

**Table 3:** Antigenic, allergenic, toxic, and physicochemical assessments of the protein translated from the mRNA vaccine-encoded peptide.

Property	Measurement	Indication
Total number of amino acids	488	Appropriate
Molecular weight	54603.7	Appropriate
Formula	C2393H3758N694O728S22	一
Theoretical pI	9.27	Acidic
Total number of negatively charged residues (Asp + Glu)	52	一
Total number of positively charged residues (Arg + Lys)	70	一
Total number of atoms	7595	一
Instability Index (II)	47.94	Stable
Aliphatic Index (A.I)	60.57	Thermostable
Grand average of hydropathicity (GRAVY)	-0.776	Hydrophilic
estimated half-life	30 hours (mammalian reticulocytes, *in vitro*)	Stable
Antigenicity (using VaxiJen)	0.7284	Antigenic
Antigenicity (using ANTIGENpro)	0.933854	Antigenic
Allergenicity (using AllerTop 2.0)		Non-allergenic
Toxicity (ToxinPred)		Non-toxic

### 3.9 In silico immune response simulation against the vaccine

**Figure 4:**
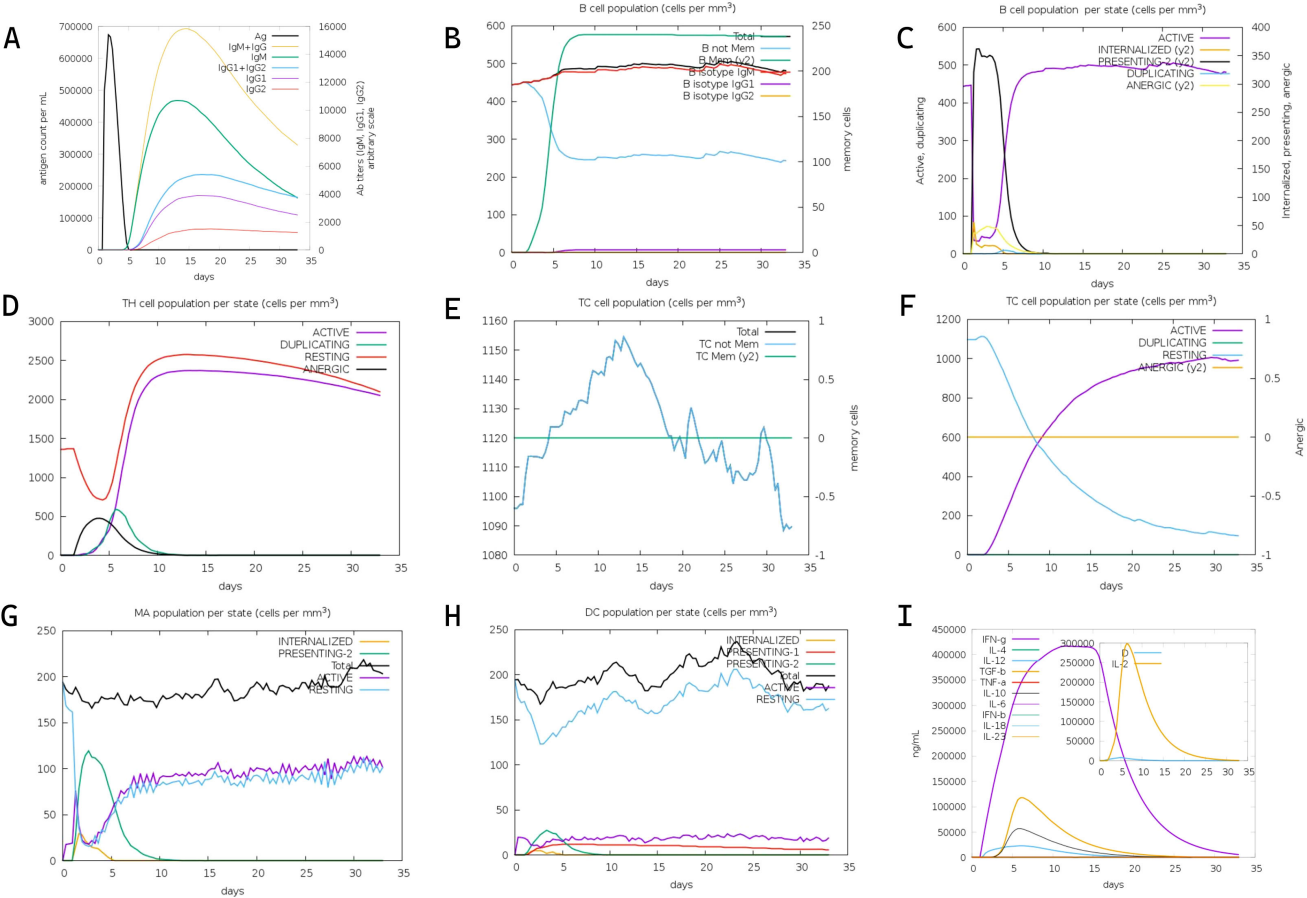
In silico immune simulation of the designed mRNA vaccine via the C-ImmSim server. **(A)** Kinetics of antigen count per nanoliter (black line, left y-axis), density of antibody-producing cells (Ab titers) per gram (gray line, right y-axis), and titers of immunoglobulins (IgM, IgM+IgG, IgG1+IgG2, IgG1, IgG2) over time post-immunization. **(B)** Total B cell population (cells per mm³, left y-axis) and memory B cell counts (right y-axis) following three vaccine doses. **(C)** Dynamics of B cell populations across distinct functional states over time. **(D)** Distribution of helper T (Th) cell populations across different states during the immune response. **(E)** Total cytotoxic T (TC) cell population (cells per mm³, left y-axis) and relative change in memory TC cells (right y-axis) over time. **(F)** Kinetics of TC cell populations across various states post-vaccination. **(G)** Dynamics of macrophage (MA) populations across different states during the simulation period. **(H)** Changes in dendritic cell (DC) populations across distinct states over time. **(I)** Production levels of cytokines and interleukins (IFN-γ, IL-4, IL-12, TGF-β, IL-10, IFN-β, IL-6, IL-1β, IL-23, IL-2) in nanograms per milliliter (ng/mL) during the 35-day simulation.

Three-dose immunization simulations (1,000 vaccine units per dose) conducted via the C-IMMSIM platform revealed coordinated adaptive immune responses (**Figure 4**). Primary analysis demonstrated predominant IgM over IgG titers post-initial vaccination, with significant immunoglobulin amplification following booster doses (**Figure 4A**). Sustained antibody elevation post-antigen clearance suggests established immunological memory, enabling rapid anamnestic responses upon antigen re-exposure. T lymphocyte profiling showed progressive expansion of both activated and resting helper T cell populations, stabilizing at elevated levels within 30 days post-immunization (**Figures 4D, E**). Peak T helper cell proliferation occurred at day 10, coinciding with macrophage activation maxima (days 5-10). Resting macrophage populations initially declined (days 0-2) before rebounding through monocyte differentiation (day 3 onward). These data indicate successful antigen presentation and lymphocyte priming. The simulation data collectively confirm the mRNA vaccine’s capacity to orchestrate robust humoral and cellular immunity against CRC.

### 3.10 Codon optimization of the mRNA vaccine construct

To maximize translational efficiency of the mRNA vaccine construct, we implemented a comparative codon optimization strategy employing five algorithms: LinearDesign, JCat, OPTIMIZER, Gensmart, and ExpOptimizer. The optimized nucleotide sequences and corresponding metrics—Codon Adaptation Index (CAI), GC content, and minimum free energy (MFE)—are detailed in **Table 4**.

**Table 4:** Comparison of five codon optimization methods for mRNA vaccine sequences: Evaluation via CAI (Codon Adaptation Index, reflects translation efficiency), GC content (impacts mRNA stability and immunogenicity), and mRNA Secondary Structure MFE(minimum free energy, indicates mRNA secondary structure stability).

Optimization method	CAI	GC	MFE
Jcat+RNAFold	0.96	67.43%	-651.20 kcal/mol
Gensmart+RNAFold	0.92	57.68%	-596.90 kcal/mol
LinearDesign	0.75	57%	-1193.70 kcal/mol
ExpOptimizer+RNAFold	0.81	52.25%	-564.30 kcal/mol
Vectorbuilder+RNAFold	0.92	56.88%	-617.10 kcal/mol

All algorithms except LinearDesign achieved comparable CAI values of approximately 0.8 (range: 0.81-0.96), while maintaining optimized GC content within the preferred human codon usage optimum (52.25-67.43%). Among conventional approaches, JCat demonstrated superior performance in both CAI (0.96) and GC content (67.43%). However, LinearDesign exhibited exceptional thermodynamic stability with an MFE of -1193.70 kcal/mol, exceeding other algorithms by more than 2.1-fold. Considering the critical importance of mRNA structural stability *in vivo*, we selected LinearDesign-optimized sequence for further development, thereby ensuring sustained antigen presentation and robust immunogenicity in human hosts.

### 3.11 Secondary structure validation and stability analysis of mRNA vaccine

**Figure 5:**
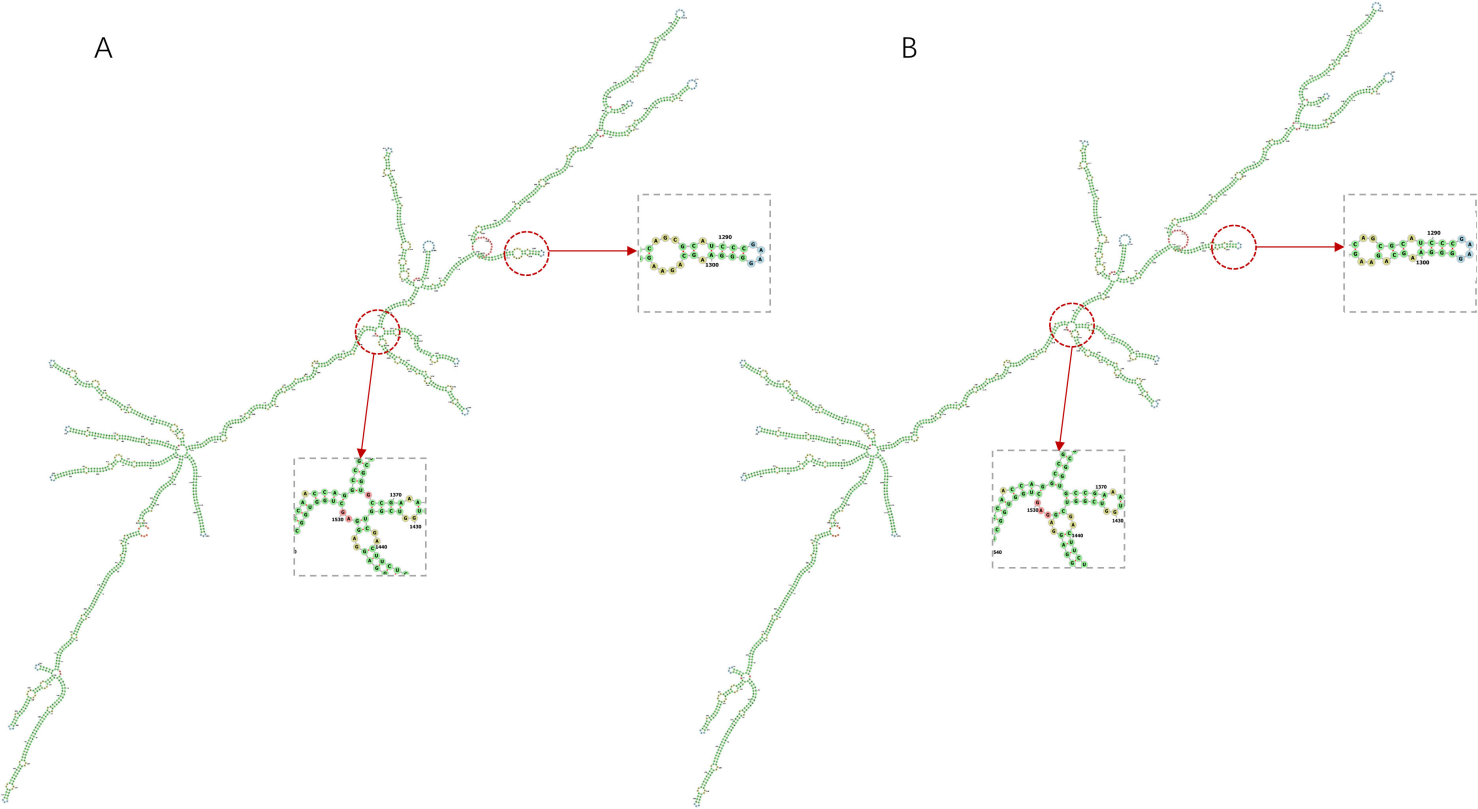
Prediction of the secondary structures of mRNA vaccine construct by two methods and their subtle differences in structural composition. **(A)** The secondary structure of the mRNA vaccine construct predicted by LinearDesign. **(B)** The secondary structure of the mRNA vaccine construct predicted by RNAfold.

The mRNA vaccine construct’s secondary structure was computationally validated through a dual-platform approach: 1) LinearDesign’s constraint programming framework predicted MFE conformations, and 2) RNAfold’s partition function algorithm (ViennaRNA Package 2.6) provided comparative thermodynamic profiling (**Figure 4**). LinearDesign yielded an MFE of -1193.70 kcal/mol, while RNAfold analysis revealed enhanced stability (MFE = -1233.87 kcal/mol). The subtle differences in the secondary structures of the sequences obtained by the two optimization methods can be seen in **Figure 5**. This convergence between distinct computational methodologies (<3.4% MFE variance) confirms exceptional structural stability. The robust thermodynamic profile correlates with enhanced vaccine efficacy through three mechanisms: 1) Reduced secondary structure interference with translational initiation complexes, 2) Increased nuclease resistance via compact folding motifs, and 3) Maintenance of epitope codon optimality under physiological temperature fluctuations (37 ± 2°C). These features collectively ensure sustained antigen expression critical for eliciting durable immune responses.

### 3.12 Molecular docking validation of TLR interaction

**Figure 6:**
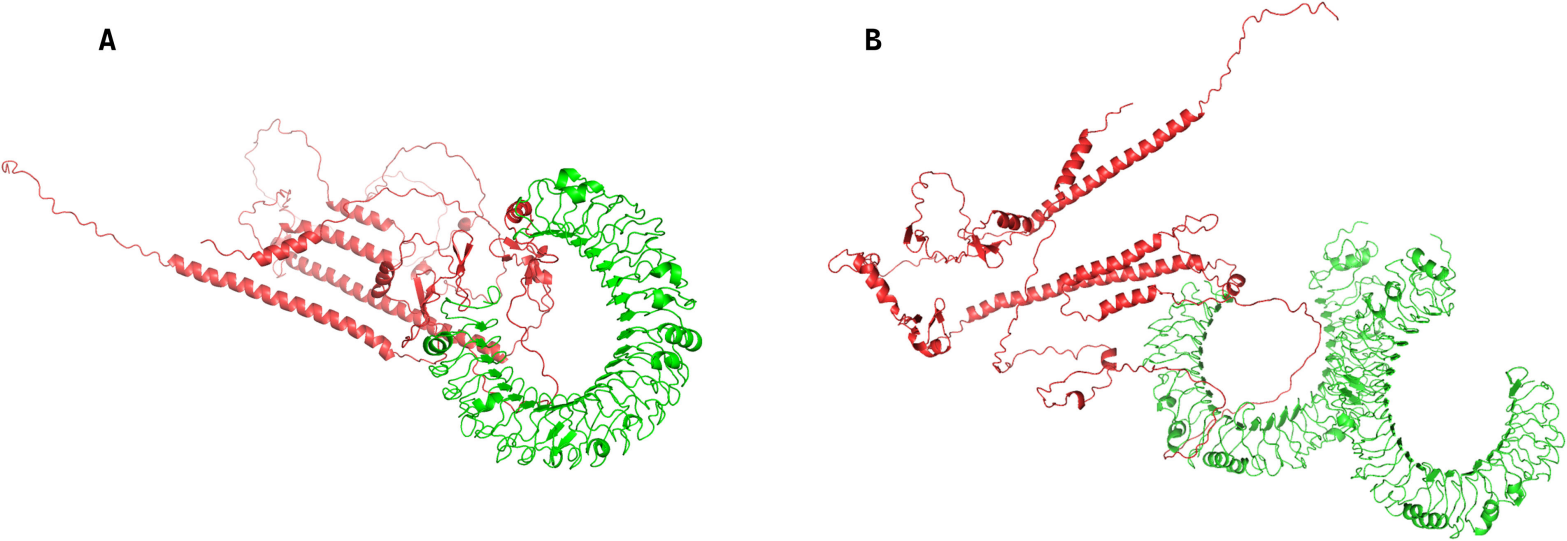
Protein-peptide docking complexes of the vaccine-encoded epitope with human Toll-like receptors, generated via ClusPro 2.0. **(A)** Docked complex between the vaccine-encoded peptide and Toll-like receptor 3 (TLR3; PDB ID 1ZIW). **(B)** Docked complex between the vaccine-encoded peptide and Toll-like receptor 4 (TLR4; PDB ID 3FXI).

Protein-peptide docking between the vaccine-encoded epitope structure and human Toll-like receptor 3 (TLR3; PDB ID 1ZIW) and Toll-like receptor 4 (TLR4; PDB ID 3FXI)was performed using ClusPro 2.0 (https://cluspro.org/), the peptide sequences produced after the translation of the mRNA vaccine are predicted by AlphaFold2 (40). with the highest-ranked TLR3-vaccine complex demonstrating a binding energy of -1378.8 kcal/mol and the highest-ranked TLR4-vaccine complex’s binding energy of -1581.2 kcal/mol (**Supplementary Table S4**, **Figure 6**). These results validate the vaccine construct’s capacity to engage TLR3 and TLR4-mediated endosomal sensing pathways, initiating robust dendritic cell maturation and subsequent Th1-polarized adaptive immunity–critical features for eliciting durable antitumor responses in colorectal cancer.

## 4 Discussion

Colorectal cancer (CRC), defined as cancer of the colon or rectum, has emerged as a persistent global health burden over the past four decades, ranking as the third most prevalent neoplastic disease worldwide (41). According to GLOBOCAN 2020 estimates by the World Health Organization’s International Agency for Research on Cancer (IARC), CRC accounts for approximately 1.93 million new cases and 935,000 deaths annually, with disproportionate incidence rates in industrialized nations (42). The clinical success of Sipuleucel-T, an autologous dendritic cell vaccine approved by the FDA in 2010 for metastatic castration-resistant prostate cancer, demonstrated the therapeutic potential of cancer vaccines and catalyzed paradigm-shifting advancements in tumor immunology (43). Cancer vaccines predominantly utilize tumor-associated antigens (TAAs) and tumor-specific antigens (TSAs) to activate antitumor immunity, enabling targeted elimination of malignant cells through immunological surveillance mechanisms (44). In this study, we engineered a multi-epitope mRNA vaccine targeting CRC by integrating six TSAs with validated oncogenic roles.

Cytotoxic CD8+ T cells play a pivotal role in tumor eradication, with clinical studies demonstrating that tumor-infiltrating CD8+ T cell density correlates with improved survival outcomes (45). Our vaccine strategy therefore prioritizes sustained activation of tumor-specific CD8+ T cells through dual mechanisms: a) Circumvention of T cell exhaustion via epitope optimization, and b) Synergistic engagement of B cell-mediated antitumor responses. Emerging evidence confirms B cells’ multifaceted roles in cancer immunity, including cytokine-mediated enhancement of CD8+ T cell cytotoxicity, granzyme B secretion for direct tumor lysis, and antibody-dependent cellular phagocytosis (46),Following epitope selection, molecular docking simulations validated robust binding affinities between prioritized epitopes and their cognate HLA alleles (**Supplementary Table S2**). The endogenous antigen processing pathway ensures cytosolic peptides complex with HLA class I molecules for CD8+ T cell recognition, a mechanism critical for eliminating malignant cells (47). Our docking results demonstrated exceptional interface stability (I_sc < -100 kcal/mol), with key epitopes like MSDTEEQEY achieving complete accommodation within HLA-A*01:01’s binding groove (**Figure 1F**).

Population coverage analysis revealed geographical immunogenic efficacy disparities, with peak coverage in Europe (89.13%) and North America (84.69%) versus reduced efficacy in Central America (15.29%) (**Table 1**). This heterogeneity reflects regional HLA allele distribution patterns, necessitating future iterations incorporating population-specific HLA haplotypes for global applicability.

Leveraging the degeneracy of the genetic code, we performed a systematic comparative analysis of five codon optimization algorithms to maximize translational efficiency and structural stability. Optimization prioritized the minimum free energy (MFE) of mRNA secondary structures as the critical stability determinant, with LinearDesign achieving superior performance (MFE = -1193.70 kcal/mol) through constraint-based programming (**Table 4**). Post-assembly bioinformatic characterization confirmed the vaccine construct’s antigenicity (VaxiJen score: 0.7284), non-allergenicity, and thermostability (instability index: 47.94), while C-IMMSIM immune simulations predicted robust lymphocyte activation (**Figure 3**). Molecular docking with Toll-like receptor 3 demonstrated exceptional binding affinity (-1378.8 kcal/mol, **Supplementary Table S4**).

Although this study has completed the design of the CRC vaccine, two points still need further research and discussion: 1) Will the antigen ordering within the vaccine construct affect the vaccine’s efficacy? 2) Can immune simulations outcomes accurately predict *in vivo* immune responses?

Among these questions, the potential impact of antigen ordering on vaccine efficacy warrants in-depth exploration, especially, for multi-antigen mRNA vaccines against CRC, where the sequence arrangement of individual antigens within the mRNA construct not only affects core antigen properties like expression and folding but also directly shapes immunogenicity and overall vaccine performance. Firstly, antigen order may influence its immunogenicity. Elena et al. (48) designed a combined influenza/COVID-19 mRNA vaccine. When the SARS-CoV-2 RBD antigen was linked to the N-terminus of the trivalent influenza antigens, the immune responses against influenza B and H3N2 antigens decreased significantly. In contrast, when the SARS-CoV-2 RBD antigen was positioned at the C-terminus, the immune responses against all influenza antigens were comparable to those induced by the trivalent influenza antigens alone. This finding indicates that antigen order may affect protein folding or antigen accessibility. Secondly, antigen order may impact its expression efficiency and cellular localization. Fang et al. (49) developed a monkeypox mRNA vaccine. The results showed significant differences in the cell surface display rates of different antigens. This suggests that the position of an antigen in the tandem structure, together with its inherent structural characteristics, collectively determines its final localization. In turn, this may affect the intensity of B cell receptor recognition and antibody responses. Interestingly, Fang et al. proposed a modular vaccine platform (MVP) in another study (50) to enhance the immunogenicity of antigens in mRNA vaccines. In that study, they pointed out that the immune responses to individual antigens in multi-antigen mixed vaccines were relatively independent, with no obvious inter-antigen interference observed. Nevertheless, the inherent cell surface trafficking capacity of different antigens varied significantly. Therefore, the order of antigens in the vaccine is less important than whether the antigens are appropriately modified. Currently, we are developing the second version of the mRNA vaccine design workflow, and based on the above conclusions, we believe that it is highly necessary to conduct further analysis and discussion on the sequence arrangement of antigen epitopes in current vaccines.

Regarding the second question, numerous previous studies have explored the reliability of simulated immune outcomes. For instance, Pappalardo et al. (51) simulated the immunoprophylactic effect of the Triplex vaccine against breast tumors in HER-2/neu transgenic mice. The model successfully reproduced the tumor-free survival curves of mice under four vaccination regimens (early, late, very late, and chronic). Additionally, the simulation results indicated that antibody (Ab) responses played a dominant role in controlling tumor growth, particularly in the long-term phase post-vaccination—this aligns with the conclusion from *in vivo* experiments that Th1-type antibody responses (e.g., IgG2a) are critical for long-term protection. Another study by Bonin et al. (52) simulated and validated the human immune response to the yellow fever vaccine. The model was highly consistent with clinical data in terms of antibody responses and viremia. Simulation results revealed that viral load peaked on day 5 post-vaccination, then decreased rapidly, and dropped to undetectable levels after 10 days—consistent with clinical observations. Similarly, in a study by Fan et al. (53), immune simulation was performed using C-IMMSIM platform as us. The results showed that the changes in B-cell and T-cell counts were highly consistent with the splenocyte subset proportions and antibody levels observed in *in vivo* experiments, demonstrating that immune simulation can effectively predict the activation trends of actual immune cells. The predicted increases in interferon-gamma (IFN-γ) and interleukin-4 (IL-4) were validated both *in vitro* and *in vivo*.

Although immune simulation exhibits a strong correlation with *in vivo* immune responses, immune simulation still faces numerous challenges in the translation to *in vivo* experiments. Firstly, immune simulation typically relies on existing knowledge, assumptions, and incorporate empirical datasets; however, these datasets may be biased or incomplete. Secondly, the human immune system is an extremely complex and multi-scale network, involving interactions at the molecular, cellular, tissue, organ, and population levels (54). These complex mechanisms are difficult to be fully captured and reproduced in a single simulation (55).Finally, there may be inter-individual differences in the immunogenicity (56).

To verify the reliability of our designed vaccine, we propose that subsequent experimental validations should include the following: conducting cellular uptake assays to evaluate the transfection efficiency of mRNA; performing protein expression assays to assess whether mRNA can be successfully translated into target antigens within cells; detecting the activation markers of CD4+ and CD8+ T cells via flow cytometry to evaluate the vaccine’s immunogenicity; and finally, evaluating whether the vaccine can inhibit tumor growth in mouse models.

## 5 Conclusion

In conclusion, the design of a novel multi-epitope mRNA vaccine for CRC in this study offers a promising framework for advancing research in CRC therapy.

However, it is crucial to note that further *in vitro* and *in vivo* studies are essential to confirm the findings of this study. These additional studies will be necessary to evaluate the vaccine’s safety, efficacy, and potential limitations in real-world scenarios. The high predicted antigenicity, the ability to interact with immune receptors, and the stable structure of the proposed vaccine suggest that it may be a promising approach to combat CRC. Overall, this study highlights the potential of in silico approaches for vaccine design and provides valuable insights into the development of effective vaccines against CRC.

## Data Availability

The original contributions presented in the study are included in the article/**Supplementary Material**, further inquiries can be directed to the corresponding author/s.
