# Supplementary material for: A novel multi-epitope mRNA vaccine against colorectal cancer: in silico design and immune efficacy profiling
Source: Front Immunol. 2025 Oct 23;16:1649091. doi: 10.3389/fimmu.2025.1649091 (PMC12589000; doi:10.3389/fimmu.2025.1649091)
Supplement: Supplementary file 1 [file Table1.docx]

***Supplementary Material***

**Supplementary Table S1. The cell types and amino acid sequences corresponding to the epitopes.**

| **Cell type** | **Sequence of epitope** | **Cell type** | **Sequence of epitope** |
| --- | --- | --- | --- |
| B lymphocytes | DEEASGADTSGVLDPD | CD4+ helper T lymphocytes | LLQVVYLHSNNITKV |
|  | ACCQRWYFTFNGAECS |  | TTLLDLQNNDISELR |
|  | PQSCVVDQTGSAHCVV |  | KISKIHEKAFSPLRK |
|  | IGRPRWKLLFDEIAKY |  | RDGFKGEKGECLRES |
|  | SGTQQRGRSCDVTSNT |  | GRDGFKGEKGECLRE |
|  | YNRISHAQKFRKGAGK |  | MNSTINIHRTSSVEG |
| CD8+ cytotoxic T lymphocytes | DPDSVTPTY |  | FVSSMGSGNPAPGGV |
|  | KLQKLYISK |  | GFVSSMGSGNPAPGG |
|  | KQKAQLRQR |  | VSSMGSGNPAPGGVC |
|  | IPKGKQKAQL |  | IQKIIGEKYHALNSR |
|  | MRPGAPGPLW |  | KPAEFTQHKFVKICM |
|  | VMYRGRCRK |  | RWKLLFDEIAKYNRG |
|  | SPFEESLNY |  | NCPYVHNPAQIDTDN |
|  | QANFPQTWLW |  | DNCPYISNANQADHD |
|  | SVDFSGTFY |  | TAQLKQDGKSRGTLL |
|  | RVSNDNQFLW |  | SEKFDLMAKLKQQKY |
|  | MSDTEEQEY |  | FDLMAKLKQQKYEIN |
|  | KEEEELVAL |  | QQRFRTEKERERQAK |

**Supplementary Table S2. CTL epitopes, HTL epitopes, and the subtypes of their corresponding HLA alleles.**

| **protein_name** | **CTL epitopes** | **MHC I binding alleles** | | **HTL epitopes** | **MHC II binding alleles** |
| --- | --- | --- | --- | --- | --- |
| BGN | DPDSVTPTY | HLA-B*35:01 | LLQVVYLHSNNITKV | | HLA-DRB1*01:01 |
|  | KLQKLYISK | HLA-A*03:01 | TTLLDLQNNDISELR | | HLA-DRB4*01:01 |
|  |  |  | KISKIHEKAFSPLRK | | HLA-DPA1*01:03/DPB1*02:01 |
| CTHRC1 | KQKAQLRQR | HLA-A*31:01 | RDGFKGEKGECLRES | | HLA-DRB1*01:01 |
|  | IPKGKQKAQL | HLA-B*07:02 | MNSTINIHRTSSVEG | | HLA-DRB1*15:01 |
|  |  |  | PAPSSASEIPKGKQK | | HLA-DQA1*01:02/DQB1*06:02 |
| FSTL3 | MRPGAPGPLW | HLA-B*53:01 | FVSSMGSGNPAPGGV | | HLA-DQA1*05:01/DQB1*03:01 |
|  | VMYRGRCRK | HLA-A*03:01 | GFVSSMGSGNPAPGG | | HLA-DQA1*05:01/DQB1*03:01 |
|  |  |  | VSSMGSGNPAPGGVC | | HLA-DQA1*05:01/DQB1*03:01 |
| NOX4 | SPFEESLNY | HLA-B*35:01 | IQKIIGEKYHALNSR | | HLA-DPA1*01:03/DPB1*02:01 |
|  | QANFPQTWLW | HLA-B*57:01 | KPAEFTQHKFVKICM | | HLA-DPA1*01:03/DPB1*02:01 |
|  |  |  | RWKLLFDEIAKYNRG | | HLA-DRB1*03:01 |
| THBS2 | SVDFSGTFY | HLA-A*01:01 | NCPYVHNPAQIDTDN | | HLA-DRB3*02:02 |
|  | RVSNDNQFLW | HLA-B*57:01 | DNCPYISNANQADHD | | HLA-DRB3*02:02 |
|  |  | HLA-B*58:01 | TAQLKQDGKSRGTLL | | HLA-DRB1*03:01 |
| TNNT1 | MSDTEEQEY | HLA-A*01:01 | SEKFDLMAKLKQQKY | | HLA-DRB1*11:01 |
|  | KEEEELVAL | HLA-B*40:01 | FDLMAKLKQQKYEIN | | HLA-DRB1*12:01 |
|  |  |  | QQRFRTEKERERQAK | | HLA-DRB5*01:01 |

**Supplementary Table S3. The docking results of some CTL epitopes with their corresponding HLA alleles. The main contributor to the Total_Score index comes from the folding energy of the monomer, and the I_sc index represents the interaction energy across the interaction interface.**

| **Type of T lymphocyte** | **Epitope** | **HLA alleles** | **PDB ID of MHC allele** | **Total Score (REU)** | **I_sc （REU）** |
| --- | --- | --- | --- | --- | --- |
| CTL | DPDSVTPTY | HLA-B*35:01 | 8EMF | -1328.4 | -24.205 |
|  | IPKGKQKAQL | HLA-B*07:02 | 7LG0 | -1186.0 | -24.431 |
|  | VMYRGRCRK | HLA-A*03:01 | 7L1C | -1123.7 | -28.003 |
|  | SPFEESLNY | HLA-B*35:01 | 4LNR | -1259.3 | -36.083 |
|  | RVSNDNQFLW | HLA-B*57:01 | 5VUD | -1207.8 | -40.581 |
|  | MSDTEEQEY | HLA-A*01:01 | 6MPP | -1094.8 | -115.51 |

**Supplementary Table S4. vaccine-TLR3 docking result**

| Receptor | Representative | Weighted Score |
| --- | --- | --- |
| TLR3 (1ZIW) | Center | -1378.8 |
|  | Lowest Energy | -1378.8 |
| TLR4 (3FXI) | Center | -1508.5 |
|  | Lowest Energy | -1581.2 |


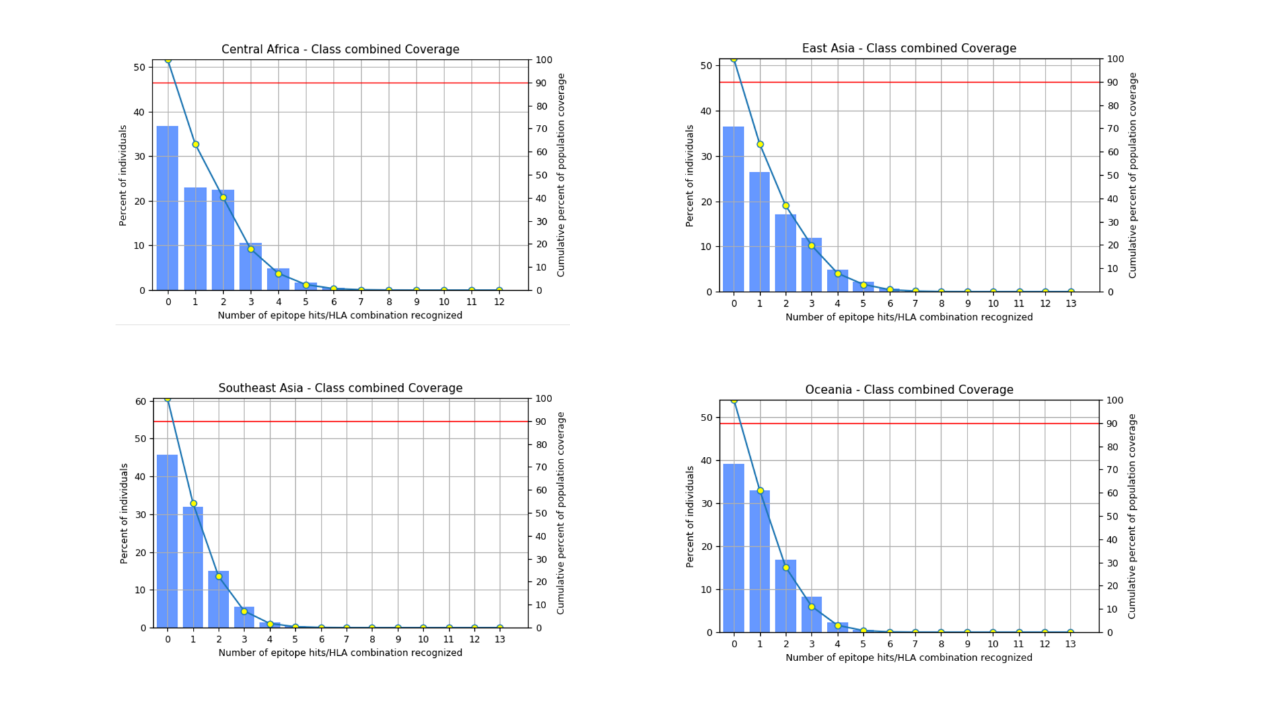


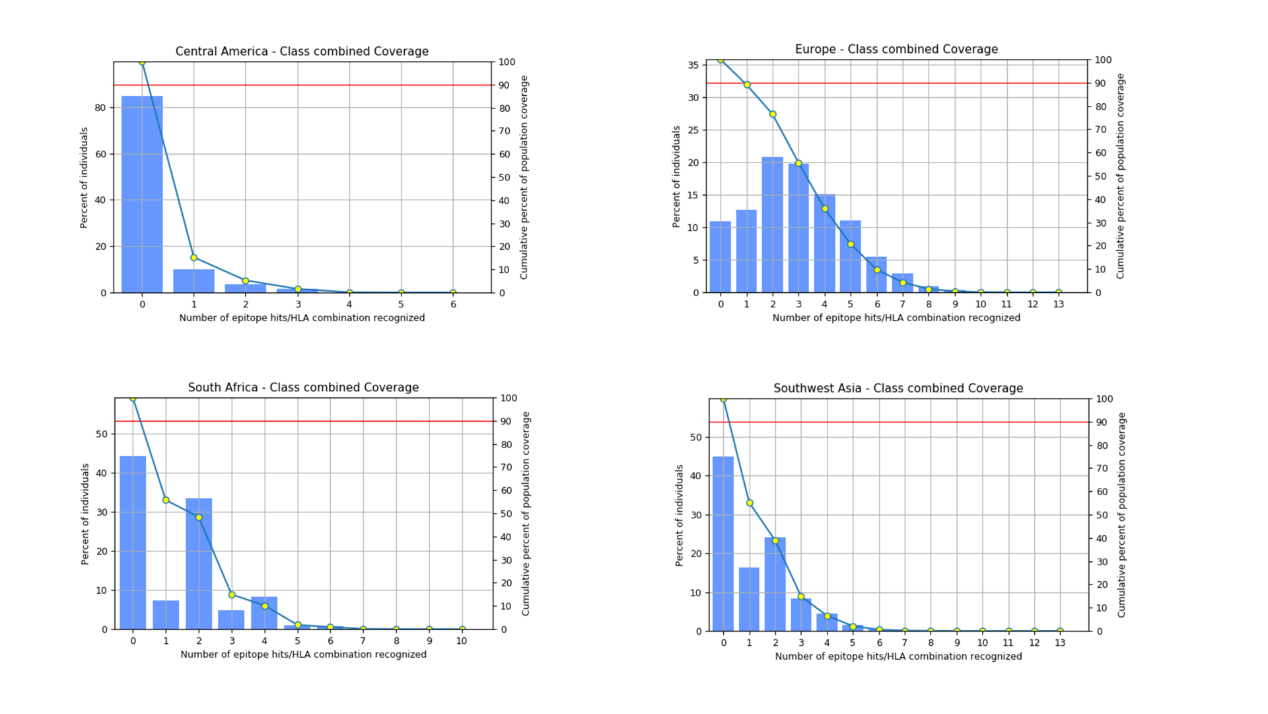


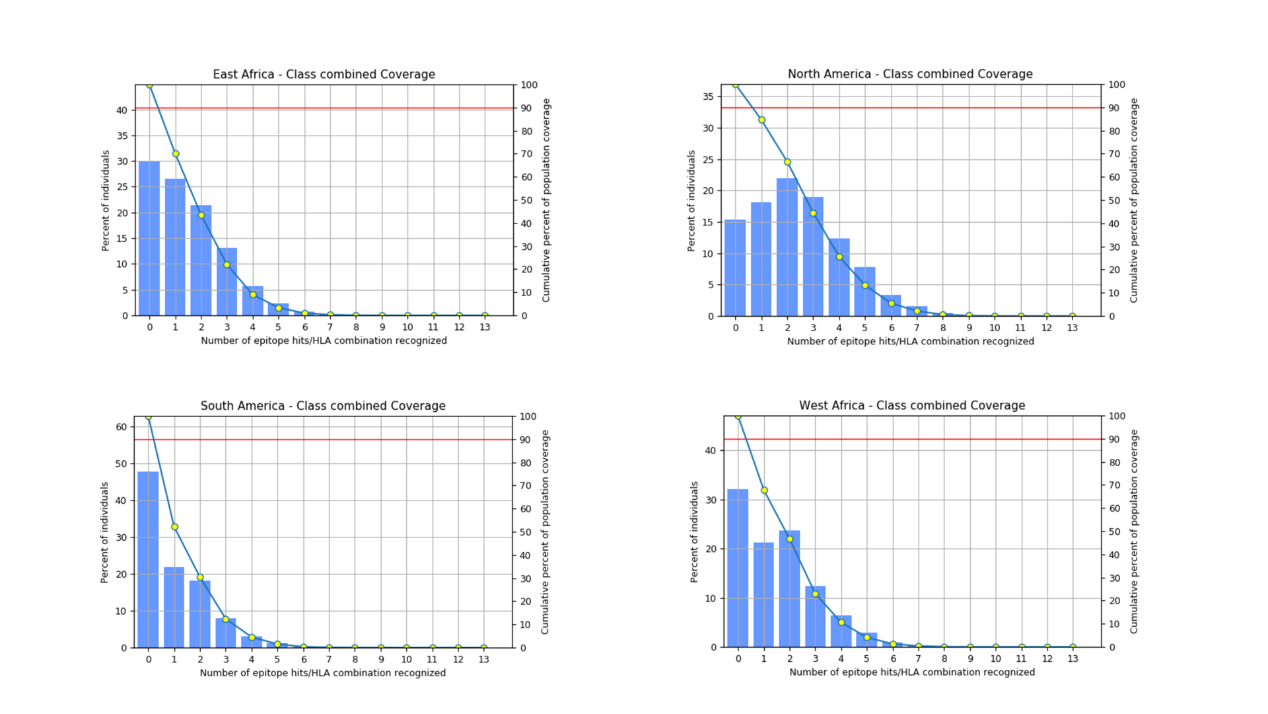


**Supplementary Figure S1. Prediction of population coverage**
